# Supplementary material for: Availability, prices and affordability of essential medicines for treatment of diabetes and hypertension in private pharmacies in Zambia
Source: PLoS One. 2019 Dec 13;14(12):e0226169. doi: 10.1371/journal.pone.0226169 (PMC6910693; doi:10.1371/journal.pone.0226169)
Supplement: S1 Table — (DOCX) [file pone.0226169.s001.docx]

**S1 Table. Median unit prices (in 2019 US-Dollars) of surveyed antidiabetics and antihypertensives.**

|  | Province | Central | | Copperbelt | | Lusaka | |  |
| --- | --- | --- | --- | --- | --- | --- | --- | --- |
|  | **Medicine** | **OBP** | **LPG** | **OBP** | **LPG** | **OBP** | **LPG** | **IRP** |
| Diabetes | Glibenclamide 5mg | 0.21 | 0.03 | 0.33 | 0.3 | 0.30 | 0.03 | 0.0061 |
|  | Gliclazide 80mg | - | 0.09 | - | 0.12 | - | 0.08 | 0.0517 |
|  | Glimepiride 2mg | - | 0.17 | 0.65 | 0.27 | 0.72 | 0.13 | 0.0425 |
|  | Insulin 30/70 soluble/isophane | 14.36 | 9.40 | 14.36 | 6.69 | 10.87 | 8.53 | 0.5075 |
|  | Insulin Intermediate-acting | - | 5.85 | 14.36 | 6.69 | 10.87 | 6.81 | 0.5899 |
|  | Insulin Short-acting | 14.36 | 6.27 | 14.42 | 6.69 | 10.87 | 6.52 | 0.6289 |
|  | Metformin 500mg | - | 0.04 | 0.17 | 0.03 | 0.17 | 0.04 | 0.0159 |
|  | **Median price all surveyed antidiabetics** | 14.36 | 0.17 | 7.50 | 0.3 | 5.795 | 0.13 | - |
| Hypertension | Amlodipine 5mg | - | 0.03 | 1.00 | 0.04 | 0.79 | 0.04 | 0.0168 |
|  | Amlodipine 10mg | - | 0.05 | 1.42 | 0.06 | 1.42 | 0.05 | 0.0168 |
|  | Atenolol 50mg | - | 0.03 | 0.48 | 0.03 | 0.44 | 0.04 | 0.0394 |
|  | Bendroflumethiazide 5mg | - | 0.12 | - | 0.12 | - | 0.11 | 0.0157 |
|  | Bisoprolol 5mg | - | 0.10 | - | 0.20 | 0.6 | 0.14 | 0.0969 |
|  | Captopril 25mg | - | 0.03 | - | 0.04 | - | 0.04 | 0.0261 |
|  | Carvedilol 6.25mg | - | 0.16 | 0.84 | 0.24 | 0.84 | 0.20 | 0.0586 |
|  | Carvedilol 25mg | - | - | 0.92 | - | 0.92 | 0.23 | 0.0466 |
|  | Enalapril 5mg | - | 0.03 | - | 0.04 | - | 0.04 | 0.0110 |
|  | Enalapril 10mg | - | 0.04 | - | 0.04 | - | 0.04 | 0.0469 |
|  | Enalapril 20mg | - | 0.05 | - | 0.05 | - | 0.05 | 0.0121 |
|  | Hydralazine 25mg | - | - | - | 0.20 | - | 0.22 | 0.0432 |
|  | Hydralazine Injection | - | - | - | 4.18 | - | 1.88 | 4.0961 |
|  | Hydrochlorothiazide 25mg | - | 0.03 | - | 0.08 | - | 0.06 | 0.0046 |
|  | Hydrochlorothiazide 50mg | - | 0.06 | - | 0.04 | - | 0.04 | 0.0052 |
|  | Lisinopril 5mg | - | - | 0.43 | 0.12 | 0.37 | 0.09 | 0.0637 |
|  | Lisinopril 10mg | - | - | 0.43 | 0.13 | 0.37 | 0.10 | 0.0939 |
|  | Lisinopril 20mg | - | - | 0.51 | 0.25 | 0.37 | 0.20 | 0.0247 |
|  | Losartan 50mg | - | 0.10 | - | 0.10 | - | 0.10 | 0.1222 |
|  | Metoprolol 100mg | - | - | 1.84 | - | 1.84 | 0.14 | 0.0219 |
|  | Nifedipine 10mg | - | 0.03 | - | 0.03 | 1.04 | 0.04 | 0.0353 |
|  | Nifedipine 20mg SR | - | 0.05 | - | 0.04 | - | 0.05 | 0.0247 |
|  | Propranolol 10mg | - | 0.03 | 0.25 | - | 0.21 | 0.1 | 0.0388 |
|  | Propranolol 40mg | - | 0.04 | 0.33 | 0.03 | 0.26 | 0.04 | 0.0073 |
|  | Verapamil 50mg | - | 0.04 | - | 0.31 | - | 0.1 | 0.0499 |
|  | **Median all surveyed antihypertensives** | - | 0.04 | 0.51 | 0.07 | 0.6 | 0.09 | - |

IRP: international reference price; LPG: lowest-priced generic; mg: milligram; n: number of pharmacies surveyed; OBP: original brand product; sol./isophane: soluble/isophane; SR: sustained release; %: percent.
